# The Effect of Mobile eHealth Education to Improve Knowledge, Skills, Self-Care, and Mobile eHealth Literacies Among Patients With Diabetes: Development and Evaluation Study

**DOI:** 10.2196/42497

**Published:** 2023-12-06

**Authors:** Sophie Huey-Ming Guo, Jiun-Lu Lin, Hung-Chun Hsing, Chun-Chuan Lee, Shih-Ming Chuang

**Affiliations:** 1 Department of Nursing Mackay Medical College New Taipei Taiwan; 2 Division of Endocrinology and Metabolism Mackay Memorial Hospital Taipei Taiwan; 3 Department of Nursing Hsinchu Cathay General Hospital HsinChu Taiwan

**Keywords:** mobile eHealth technology, mHealth literacy, eHealth literacy, diabetes, HbA1c, self-care behavior

## Abstract

**Background:**

The promotion of mobile health (mHealth) and eHealth technologies as tools for managing chronic diseases, particularly diabetes mellitus, is on the rise. Nevertheless, individuals with diabetes frequently face a literacy gap that hinders their ability to fully leverage the benefits offered by these resources. Enhancing technology literacy to facilitate the adoption of mobile eHealth services poses a significant challenge in numerous countries.

**Objective:**

This study aims to develop an educational mobile eHealth literacy (eHL) program for patients with diabetes and to evaluate its effect on patients’ outcomes.

**Methods:**

This study designed a mobile eHL education program comprising 2 modules specifically tailored for individuals with type 2 diabetes (T2D). These modules focused on guiding participants through the process of effectively navigating reliable health websites and utilizing diabetes-related apps. Using a pre- and posttest experimental design, the study featured an intervention group and a control group. Participants were recruited from 3 outpatient departments in hospitals, and assessments were conducted both before and after the intervention, along with a follow-up measure at the 3-month mark. The evaluation encompassed sociodemographic characteristics, computer and internet proficiency, mobile app usage, mobile eHL, and patient outcomes such as self-care behaviors and glycated hemoglobin (HbA_1c_) levels.

**Results:**

The analysis included a total of 132 eligible participants. Significant differences were observed in the mean scores of knowledge (*P*<.001) and skills (*P*<.001) related to computers, the web, and mobile devices at the initiation of the study and after the intervention. During the 3-month follow-up, the findings indicated a significant improvement in mobile eHL (*t*_114_=3.391, *P*=.001) and mHealth literacy (mHL, a subconcept of mobile eHL; *t*_114_=3.801, *P*<.001) within the intervention group, whereas no such improvement was observed in the control group. The chi-square values from the McNemar test underscored that individuals with uncontrolled diabetes (HbA_1c_≥7%) in the intervention group exhibited more improvement compared with the control group. The generalized estimating equations model unveiled a significant difference in the change of general mHL in the intervention group (β=1.91, *P*=.047) and self-care behavior in the control group from T0 to T2 (β=–8.21, *P*=.015). Despite being small, the effect sizes for mobile eHL (*d*=0.49) and HbA_1c_ (*d*=0.33) in the intervention group were greater than those in the control group (*d*=0.14 and *d*=0.16, respectively).

**Conclusions:**

The implementation of a mobile eHL education intervention demonstrates a positive influence on the familiarity of patients with T2D regarding health technology, leading to favorable glycemic outcomes. While additional studies are warranted for a more comprehensive understanding, this program emerges as a promising solution for enhancing patients’ uptake of digital health technology.

## Introduction

### Background

In the digital age, health technology has emerged as a promising and empowering tool to bridge the gap between the needs of chronic patients and the capabilities of health care systems [1,2]. Diabetes is one of the most prevalent chronic conditions, affecting nearly 1 in 10 adults globally [3]. Meta-analyses and reviews have consistently shown that the adoption of eHealth and mobile health (mHealth) technology proves to be a fruitful strategy in simultaneously enhancing various outcomes related to diabetes [4-7]. These outcomes span both short- and long-term effects, encompassing improved disease knowledge and skills, enhanced self-care behavior, and measurable indicators such as glycated hemoglobin (HbA_1c_).

Recent data have revealed that over 70% of individuals engage in online searches and utilize mobile apps [8,9]. However, this widespread usage does not necessarily translate into a comprehensive embrace of health technology [10-14]. In a specific study, it was observed that 75% of adults without diabetes owned smartphones, and among them, approximately 30% used health apps. By contrast, among adults with type 2 diabetes (T2D), 42% owned smartphones, with only 14% of this group utilizing health apps [9]. In another survey, this figure was reported to decrease even further, reaching as low as 2% [15]. Clearly, individuals with diabetes exhibit lower levels of engagement with health technology, with a primary factor being a lack of awareness regarding the existence of health apps [9]. Additionally, routine patient education tends to concentrate on the disease itself, overlooking the potential benefits of health technology, despite health care professionals expressing the desire to enhance disease care through the deployment of eHealth and mobile apps [12,15,16]. This prompts the question of how patients with diabetes can be better equipped with the knowledge and comfort to effectively utilize digital tools.

Our study endeavors to empower patients with T2D by enhancing their understanding of the utilization of health websites and mHealth apps, with the ultimate goal of improving health outcomes. Consequently, the first phase of our research involved the development of an educational toolkit designed to enlighten patients about mobile eHealth. Next, our study delved into the impact of the educational toolkit on patients’ short-term outcomes and conducted a comparative analysis of the long-term outcomes after a 3-month period. The underlying hypothesis posits that individuals who undergo mobile eHealth literacy (eHL) education interventions would exhibit more favorable outcomes.

### People Need Help Accessing eHealth Resources and mHealth Apps

Literacy-related disparities contribute to unequal access to eHealth resources and variations in health app utilization among patients with chronic conditions. The information available on health websites or social media may not always undergo verification by health professionals, leading to a mix of accurate and inaccurate content [17,18]. From the perspective of patients, online health-related information can be exceedingly intricate and perplexing [19-22]. Individuals with diabetes who possess lower levels of mHL and eHL may face challenges in comprehending and accessing eHealth information as well as utilizing mHealth apps [15,16].

The literature indicates that individuals who use health apps generally tend to be younger, have higher levels of education, and report engaging in more physical activity [9]. Moreover, users who perceived the app as having a significant impact on their health were observed to be in better health overall, exhibited higher levels of eHL, and actively utilized the app to implement behavior-changing techniques [10]. In other words, individuals who are not users of mHealth apps often belong to older age groups or have lower levels of education, placing them in a comparatively disadvantaged position [2]. Furthermore, a usability evaluation uncovered that patients could only independently accomplish 43% of tasks on their mHealth apps, highlighting the potential challenges in user interaction and navigation [23].

Additional factors contributing to challenges in the implementation of mHealth apps are a potential disconnect between the apps and the users’ capabilities [13,24], as well as issues related to the suboptimal quality of diabetes apps [25]. Likewise, a study that assessed 101 apps revealed that a majority of health apps did not align with the mHealth literate design strategies outlined by the Institute of Medicine [26]. This is evident in the fact that certain technology-driven interventions demonstrate insignificant benefits and, in some cases, even exhibit negative impacts [5,27,28]. Recognizing the concerns of health care providers about the potential increase in workload associated with the integration of mobile eHealth technology into primary care services [12], a more effective solution becomes imperative.

### Literacies of mHealth and eHealth

Mobile eHL has 2 vital main subconcepts: eHL and mHL. eHL involves the capacity to assess health information from electronic sources and utilize the acquired knowledge to confront or resolve a health issue. It places emphasis on eHealth information rather than traditional sources, such as pamphlets and printed patient handouts. mHL is another emerging literacy that is broadly defined as the capability to use mobile devices to search, find, comprehend, assess, and apply health information when addressing or resolving health issues [29]. A recently updated mobile eHL description encompasses not only health information seeking and appraisal on mobile devices but also the competence to access mHealth apps, download them from an app store platform, and register these apps. These aspects have been validated in our previous study [15].

### Evidence Exists Regarding Mobile eHealth Intervention

Research in the realm of mobile eHL interventions is expanding, yet its comprehension among patients with chronic conditions, particularly those with diabetes, is still not well-established [30,31]. A novel blended approach, combining face-to-face and online or computer-based education, is currently in the testing phase [32]. In the digital era, the ubiquity of misinformation is a significant concern [33], and the need for vulnerable patients, such as those with diabetes, to have access to reliable sources and useful self-care tools is of vital importance to health care providers [23,34-36].

A recent protocol introduced a digital health intervention in Pakistan that leveraged digital tools, such as smartphones and the internet, to aid mothers and families during the COVID-19 pandemic [37]. In a distinct group-based eHL intervention carried out in the United States, involving 146 older adults, more favorable outcomes were observed when they were instructed by professionals using a toolkit from the National Institute on Aging (NIA). This toolkit assists older adults in navigating health information modules [31]. However, limitations arise due to the absence of mHealth considerations. Generalizing these findings to individuals with chronic diseases becomes challenging with an older adult sample. Additionally, the absence of mHealth components poses a hindrance to the broader expansion of health app usage.

On the other side, the NIA toolkit comprises well-organized content across various categories, including computer basics, internet basics, email basics, NIHSeniorHealth FAQs, a site map and search box, and an introduction to health-related websites (such as MedlinePlus, NIHSeniorHealth, Go4Life, MedlinePlus Drugs and Supplements, and the Medical Encyclopedia), among others [31]. This comprehensive toolkit empowers individuals to develop the capability to discern the reliability of online health information.

In this study, our research team utilized the meticulously organized toolkit [31] to create digital educational materials tailored specifically for the diabetic population. Additionally, we incorporated insights from studies on the literacies of mHealth and eHealth, as highlighted in our earlier research studies [15,29]. In certain aspects, these studies align with Ellen et al’s [38] recommendation to empower patients to take an active role in managing their health.

## Methods

### Study Design and Setting

The initial phase of our research project involved an investigation into mobile eHL and an exploration of its influencing factors [15]. This second part of our research project is dedicated to scrutinizing the impact of a mobile eHL intervention on improving various outcomes for patients with diabetes. The study used a quasi-experimental design, incorporating both pre- and posttests, along with a longitudinal approach. The sample size was determined by considering the disparity in eHL observed between the 2 groups in the preceding study, resulting in a calculated sample of 160 participants. Data were collected from the intervention group at 3 different time points: the pretest (baseline), the posttest (immediately after the intervention), and a follow-up at 3 months. The control group completed the baseline questionnaire and a follow-up assessment at the 3-month mark, as outlined in Table 1. The research took place in the outpatient department of endocrinology and metabolism at 3 hospitals in Taiwan, spanning from January 2017 to January 2018.

**Table 1:** Measures used and their measurement time between the 2 groups.

Scale		Baseline test (T0)			Immediate posttest (T1)			3-month follow-up (T2)	
		Interventional group	Control group	Interventional group		Control group	Interventional group		Control group
**Demographic variable**									
	Sex, age, among others	✓	✓						
	Self-rated health								
	A habit of mHealth and eHealth use								
**Part 1 test**									
	Knowledge of computer/web/mobile	✓	✓	✓					
	Skills in computer/web/mobile	✓	✓	✓					
**Part 2 test**									
	Mobile eHealth literacy^a^	✓	✓				✓		✓
	Self-care behavior	✓	✓				✓		✓
	HbA_1c_^b^	✓	✓				✓		✓

^a^Mobile eHealth literacy consists of 2 main concepts, namely, eHealth literacy and mobile health literacy.

^b^HbA_1c_: glycated hemoglobin.

### Ethics Considerations

This study received approval from the institutional review board of the designated hospitals (institutional review board approval numbers 17MMHIS003e and CGH-OP105003) and adhered to the CONSORT-EHEALTH (Consolidated Standards of Reporting Trials of Electronic and Mobile Health Applications and Online TeleHealth) guidelines during its execution. All respondents were informed that participation was voluntary, that they could leave at any time without reason, and that their choice to participate would not affect their care. All who chose to participate had to give their written consent.

### Participants

Study participants were referred by endocrinologists or certified diabetes educators. Inclusion criteria were individuals who (1) had a diagnosis of diabetes and were aged between 20 and 65 years, (2) possessed basic reading and writing abilities, (3) did not have vision defects, and (4) expressed a willingness to partake in the research study. Exclusion criteria encompassed individuals with severe vision loss, communication difficulties, or alcohol or drug abuse problems. Participants with smartphones were eligible for recruitment into the intervention group, while the control group comprised an independently recruited cohort.

Eligible patients were interviewed in the waiting rooms of the outpatient department, ensuring a safe, private, and secure environment. Our researchers provided a detailed explanation of the procedure to each participant, covering the study’s purpose, the methodology, the anticipated time required to complete the questionnaire, and how the collected data would be used. Subsequently, participants completed the questionnaires and skill performance testing. As an incentive for their participation at each stage, they were provided with a gift card valued at NT $50 (US $1.50) upon completion of the questionnaires.

### Designing the Mobile eHealth Literacy Program

The intervention took the form of a “mobile eHL program,” which included instructional materials. The fundamental components of the study comprised 8 elements of mobile eHL: traditional literacy, health literacy, information literacy, scientific literacy, media literacy, computer literacy, mobile literacy, and internet literacy. These concepts were integrated into the intervention program developed by our research team and were directly connected to the measurement. This study introduced a mobile eHealth education program consisting of 2 modules for patients with diabetes (Multimedia Appendix 1). The first module was adapted from the NIA toolkit, which comprises educational materials designed to train older adults in accessing and utilizing online health information. The criteria they use to evaluate health websites and the effectiveness of this toolkit have been validated by Xie [31]. In customizing the first module for the target populations in this study, American health websites were replaced with 3 Taiwanese health websites, including the National Educational Resource website [39], supported by the Health Promotion Administration under the Ministry of Health and Welfare. The second module incorporated information on the 3 most popular diabetes apps, along with instructions for operating mobile devices and practical exercises on using diabetes apps. To ensure content validity, the mobile eHealth modules were evaluated by 6 clinical and academic experts, receiving a content validity index score of 0.86.

The mobile eHealth modules were executed on an Apple Macintosh computer and transferred onto an iPad (Apple Inc.). These modules incorporated interactive multimedia elements, such as image and story videos, as well as interactive components such as questions and quizzes. The content was extensively designed to leverage these interactive multimedia features. The iBook Author software (Apple Inc.) was chosen for its ability to create a user-friendly interface and incorporate sophisticated interactive media. Screenshots of the mobile eHL modules are presented in Multimedia Appendices 2 and 3.

The theoretical rationale for this intervention was guided by existing literature and frameworks on eHL [40-43]. The mobile eHL intervention aimed to enhance patients’ proficiency in utilizing mobile eHealth resources, including mHealth apps, with the goal of influencing patient outcomes, particularly in terms of self-care behaviors. Upon obtaining informed consent from both groups, trained interviewers collected baseline data. The intervention participants received personalized, 1-on-1 patient education with the mobile eHealth modules from members of the research team. This involved coaching participants in the use of health websites and diabetes apps for 30-60 minutes.

Following the completion of the program by the intervention group, the posttest was promptly administered, encompassing a satisfaction survey and a test evaluating knowledge and skills related to mobile and internet usage. Approximately 3 months after the enrollment date, both groups underwent follow-up measures, which included the mobile eHL and self-care behavior questionnaires. In adherence to ethical considerations, the control group was provided with printed material from the mobile eHealth educational program after they had completed all the questionnaires during the 3-month follow-up.

### Outcomes and Measurements

#### Overview

The demographic information collected encompassed age, sex, education, health status, duration of diabetes, experience with mobile and internet use, and habits related to seeking health information online. Supplementary measures included validated assessments of mobile eHL, knowledge, and skills related to mobile app and internet use. The health outcomes, considered as dependent variables, comprised self-rated health, diabetes self-care behavior, and HbA_1c_.

#### Mobile eHealth Literacy Questionnaire

The instrument comprised 3 scales: eHL (8 items) [44], mHL (8 items) [15], and mobile eHealth preference (4 items) [15,44] (refer to Multimedia Appendix 4). First, eHL was assessed using Norman and Skinner’s eHealth Literacy Scale (eHEALS) [44], a scale that gauges perceived skills and comfort in utilizing the internet for health information and decision-making. Factorial validity and internal consistency (Cronbach α=.94) were reported.

Second, the mHL questionnaire was developed in our prior research [15], adapting elements from Norman and Skinner’s eHEALS [44] and relevant literature [29]. The inclusion of mobile skills aimed to provide a comprehensive assessment of all aspects related to using internet resources through mobile technology [45]. This section poses questions regarding perceived skills related to mHealth apps for self-management. For instance, in one of the eHEALS items—“I can tell high-quality health resources from low-quality health resources on the internet,” the wording was adapted to “I can assess the quality of health apps (quality meaning: the functionality and content of apps).”

Third, the mobile eHealth preference (mobile eHL preference) was assessed by soliciting individual opinions about mHealth and eHealth technology. An example item is “How important is it for you to be able to access health resources on the internet?” Each item in the 3 subscales is rated on a 5-point Likert scale, where 1=strongly disagree and 5=strongly agree, with higher scores indicating a greater proficiency in using mHealth technology.

The content validity index for the 3 questionnaire subscales was determined by 6 senior experts, consisting of 2 metabolism physicians, 2 dietitians, and 2 professors in the health informatics discipline. These experts were affiliated with 3 hospitals and 2 universities in Taiwan. The content validity index score was used to evaluate the relevance, clarity, and simplicity of each item. An acceptable content validity score was considered to be 0.80 or higher [46]. Face validity was assessed with 3 voluntary participants with diabetes. In our previous study [15], the Cronbach α values for eHL, mHL, and mobile eHL preference scores were .927, .927, and .847, respectively.

#### Knowledge and Skills of Mobile Technology and the Internet

The knowledge and skills questionnaires incorporate components related to the use of computers, the web, and mobile apps, which were adapted from a previous study [31]. This questionnaire was formulated to address the limitations of the eHL measure, which solely reflects individuals’ perceived performance on online tasks and lacks an objective measure [47]. The first knowledge-related test comprises 15 items, scored 1 if answered correctly and 0 if answered incorrectly. An example item is, “Try to find a pictogram meaning a place for downloading apps.” The second skills-related test consists of 10 items, with each item scored 1 if operated appropriately and 0 if operated inappropriately. Examples of items include “Please try to open a browser and connect to a health website” and “Please try to download and use a diabetes app on a mobile device.” The reliability, assessed using the Kuder-Richardson Formula 20 (KR-20), was 0.905 for the knowledge test and 0.923 for the skills test, respectively. Face validity was assessed with 3 voluntary participants with diabetes.

#### Self-Rated Health

Subjective health status, as derived by Hornby-Turner et al [48], was assessed by asking participants to respond to 3 questions, such as “How would you describe your general health, is it good, fair, or poor?” Responses ranged from very good (rating=1) to poor (rating=3). Higher scores indicated better perceived health.

#### Diabetes Self-Care Behavior Questionnaire

The Diabetes Self-Care Behavior 36-item questionnaire, developed by Parchman et al [49], evaluates the extent to which patients adhere to recommended self-care activities. For instance, participants are queried about how frequently they adhere to the recommended daily diet in a typical week. Behavior is gauged on a 5-point ordinal scale: 0=never, 1=1-3 times per week, 2=4-5 times per week, 3=more than 5 times per week, and 4=always. A higher score indicates a more frequent engagement in self-care behavior.

#### Glycated Hemoglobin

HbA_1c_ serves as a crucial indicator of glycemic control, reflecting the average blood glucose level over 3 months. The HbA_1c_ data for the study participants were obtained by reviewing electronic medical records during the enrollment period. HbA_1c_ of 7% serves as a cutoff point and a value less than 7% is considered indicative of good control. Higher levels of HbA_1c_ suggest poor glycemic control, which is associated with an elevated risk of vascular complications and death [39,50].

### Statistical Methods

The data were analyzed using the SPSS statistical software package (version 22.0; IBM Corp.). The analysis included descriptive and exploratory statistical analyses. As the knowledge and skills tests in this study are dichotomously scored, the Kuder-Richardson Formula 20 was used to present their reliability. The KR-20 is commonly used to measure the internal consistency/reliability of a test in which each question has only 2 answers: right or wrong. The value for the KR-20 ranges from 0 to 1, with higher values indicating higher reliability [51]. A generalized estimating equation analysis, *t* test (2-tailed and paired), and McNemar chi-square test were used to compare the scores at 3 time points. The McNemar chi-square test is suitable for paired nominal data. Interaction model generalized estimating equations were derived with groups, time, and a group × time interaction term entered as independent variables. A line graph was utilized to illustrate the mean differences at the pretest (T0), posttest (T1), and 3-month follow-up (T2) periods.

The effect size was calculated for clinical interpretation, providing insights into the effectiveness of an intervention [52,53]. Effect size, typically represented by a standardized measure such as Cohen *d*, is based on the differences between 2 means. The value of the effect size can be commonly interpreted as very small (*d*<0.2), small but worth noting (*d*=0.2-0.5), medium (*d*=0.5-0.8), and large (*d*≧0.8) [54,55].

The effect size of the intervention or the combined effect size illustrates the changes observed in the intervention group compared with the control group. Merely reporting statistical significance is insufficient as it fails to demonstrate the effectiveness of the intervention. In the reporting and interpretation of study results, both substantive significance (effect size) and statistical significance (*P* value) are crucial [55]. The rationale for reporting effect sizes lies in the fact that a significant *P* value indicates the efficacy of an intervention, while an effect size quantifies the extent of this efficacy. It is important to note that Cohen *d* is not linked to statistical significance. The group-effect size of pre- and postintervention changes was calculated using an online statistical tool [56].

## Results

### Participant Flow

The participant flow is outlined in Multimedia Appendix 5. Initially, 160 participants were randomly selected from the first part of the project and invited to participate in the second part of the research. Among the initial 160 participants, 4 refused to participate, and 24 were excluded due to incomplete data, leaving 132 participants eligible for the final analysis. Subsequently, the eligible participants (N=132) were randomized into either the intervention group (n=96) or the waitlist control group (n=36) after completing the baseline assessment.

### Baseline Characteristics

Table 2 outlines the demographic characteristics of the patients, revealing no significant baseline differences between both groups in any of the demographic variables.

In the intervention group, patients had an average age of 42 (SD 8.923, range 22-62) years, with 72/96 (75%) being male. A significant majority, 61/96 (64%), had an educational level of at least college or university (*P*=.53). The mean duration of T2D was 4.967 (SD 5.64, range 0-26) years. Most participants in this group reported their health status as fair. The average HbA_1c_ result was 8.14 (SD 2.15, range 5.3-14.4) mg/dL, and for 57/93 (61%) participants in this group, it exceeded 7 mg/dL.

In the control group, the mean age was 41 (SD 7.907, range 21-61) years, with 21/37 (57%) being male. About 61% (22/36) had an educational level of at least college or university. The mean duration of T2D was 4.16 (SD 3.37, range 0-14) years. The majority of participants in this group reported their health status as fair. The average HbA_1c_ result was 7.80 (SD 1.76, range 5.7-12.8) mg/dL, and for 21/36 (58%) participants in this group, it exceeded 7 mg/dL.

Technology use between the 2 groups was comparable in terms of online health information seeking and the utilization of mHealth apps, as indicated in Table 3. Among the 132 participants, 57/95 (60%) in the intervention group and 30/36 (83%) in the control group reported having searched for online health information. The chi-square test revealed significant differences (*P*=.01) in online health information seeking between the groups. However, there was no statistical difference between the groups in terms of experience with mobile and internet use.

**Table 2:** Sociodemographic characteristics of the 2 groups of participants.

Characteristics			Intervention (n=96)		Control (n=36)		Group differences, chi-square/*t* value (*df*)		*P* value
**Sex, n (%)**							3.494 (1)		.08
	Male	72 (75.0)		21 (58)					
	Female	24 (25.0)		15 (42)					
Age in years, mean (SD); range			42.08 (8.923); 22-62		41.36 (7.907); 21-61		0.748 (130)		.39
**Education level, n (%)**							1.274 (1)		.53
	High school or less	35 (36)		14 (39)					
	College or university	51 (53)		16 (44)					
	Master or PhD	10 (10)		6 (17)					
Duration of type 2 diabetes, mean (SD); range^a^			4.967 (5.64); 0-26		4.16 (3.37); 0-14		0.904^b^ (106)		.37
**Self-rated health, n (%)**							–0.756 (1)		.45
	Good	14 (15)		8 (22)					
	Fair	64 (67)		22 (61)					
	Poor	17 (18)		6 (17)					
**HbA_1c_^c^ (mg/dL), mean (SD); range**			8.14 (2.15); 5.3-14.4^d^		7.80 (1.76); 5.7-12.8^e^		0.855 (127)		.39
	<7, n (%)	36 (38)		15 (42)		1.92 (1)		.69	
	≧7, n (%)	57 (59)		21 (58)					
	7.0-8.0, n (%)	20 (21)		10 (28)					
	8.1-9.0, n (%)	14 (15)		4 (11)					
	9.1-10.0, n (%)	6 (6)		2 (6)					
	10.1-15, n (%)	17 (18)		5 (14)					

^a^Some participant values are missing.

^b^Welch *t* test.

^c^HbA_1c_: glycated hemoglobin.

^d^n=93.

^e^n=36.

**Table 3:** Participant habits regarding online health information seeking and the use of mHealth apps.

Characteristics		Intervention (n=96)	Control (n=36)		Significance, chi-square or *t* value (*df*)	*P* value	
**Searched for diabetes/health information, n (%)^a^**					6.372 (129)	.01	
	Have	57 (60.0)	30 (83)				
	Have not	38 (40.0)	6 (17)				
**Used health apps, n (%)**					1.547 (127)	.21	
	Used	4 (4.2)	0 (0)				
	Have not used	92 (96)	36 (100)				
**Use of** **technology (years), mean (SD); range**							
	Smartphone	6.62 (3.56); 0-20^b^	5.7 (3.37); 0-10^c^		1.266 (129)	.21	
	Tablet	0.553 (2.13); 0-15^d^	1.38 (3.33); 0-12		–1.305 (124)	.20	
	Computer	10.86 (8.68); 0-30	13.77 (11.63); 0-30		–1.673 (127)	.10	
**Daily use (hours), mean (SD); range**							
	Smartphone	4.39 (3.53); 0-18^b^	4.19 (3.05); 0-12^c^		2.292 (129)	.77	
	Tablet	0.17 (0.87); 0-8^d^	0.63 (1.72); 0-8^e^		–1.453 (124)	.16	
	Computer	2.77 (3.16); 0-13^b^	4.21 (4.00); 0-12^f^		–1.873 (126)	.07	

^a^Some participant values are missing.

^b^n=95.

^c^n=36.

^d^n=94.

^e^n=32.

^f^n=33.

### The Changes in Knowledge and Skills About Mobile and Internet; Preference for eHL, mHL, and Mobile eHL; HbA_1c_; and Self-Care Behavior

Mean changes within and between the 2 groups from pre- and postintervention were analyzed to assess the short- and long-term effects of the intervention. Table 4 presents the pre- and posttests (T0 and T1 measures) on the intervention day, with the T1 measure conducted exclusively in the intervention group. Following the completion of the intervention, participants in the intervention group demonstrated a significant improvement in knowledge scores (mean 0.554, SD 1.850), with a notable mean change of t_82_=2.730 (*P*=.008). Additionally, for the skill score, patients in the intervention group exhibited a significant mean change of 1.325 (SD 1.861, t_82_=6.485, *P*<.001).

**Table 4:** Differences between baseline (T0) and postintervention (T1) for the intervention group (n=96).

Variables	Baseline (T0), mean (SD)	Postintervention (T1), mean (SD)	Difference, mean (SD)	Significance	
				*t* value (*df*)	*P* value
Knowledge^a^	14.29 (2.212)	14.84 (0.862)	0.554 (1.850)	2.730 (82)	.008
Skills^a^	8.66 (1.856)	9.99 (0.428)	1.325 (1.861)	6.485 (82)	<.001

^a^Knowledge and skills in computers, web, and mobile technology, which were measured immediately after the intervention.

Table 5 presents the analyses conducted at the 3-month follow-up (T2 measure) for both groups. The paired comparisons indicated a significant improvement in mobile eHL (t_81_=3.391, *P*=.001) and its subscale, mHL (t_35_=3.801, *P*<.001), in the intervention group, whereas no significant improvement in mobile eHL was observed in the control group (t_35_=0.871, *P*=.39).

**Table 5:** Between-group differences at pretest (T0) and at the 3-month follow-up (T2).

Characteristics		Baseline (T0), mean (SD)	At 3 months (T2), mean (SD)	T0-T2 difference, mean (SD)	Significance		
					*t* value (*df*)	*P* value	
**Mobile eHL^a^**							
	Intervention	74.72 (10.938)	78.70 (9.912)	3.976 (10.617)	3.391 (81)	.001	
	Control	76.56 (12.360)	77.83 (10.219)	1.278 (8.801)	0.871 (35)	.39	
**Mobile eHL subscale: preference**							
	Intervention	14.73 (2.430)	15.32 (2.303)	0.585 (2.918)	1.816 (81)	.07	
	Control	14.69 (2.573)	15.19 (2.786)	0.500 (2.710)	1.107 (35)	.28	
**Mobile eHL subscale: eHL**							
	Intervention	30.65 (4.859)	31.72 (4.287)	1.073 (5.199)	1.869 (81)	.07	
	Control	31.806 (5.626)	32.222 (4.486)	0.4167 (3.974)	0.629 (35)	.53	
**Mobile eHL subscale: mHL**							
	Intervention	29.34 (5.776)	31.66 (4.857)	2.317 (5.519)	3.801 (81)	<.001	
	Control	30.06 (5.77158)	30.42 (4.813)	0.3611 (4.624)	0.469 (35)	.64	
**Self-care behavior**							
	Intervention	79.83 (27.166)	81.09 (26.696)	1.263 (22.079)	0.511 (81)	.61	
	Control	74.36 (26.694)	84.06 (24.305)	9.6 (2.39)	4.503 (35)	<.001	
**HbA_1c_^b,c^**							
Intervention (n=85)		8.09 (2.113)	7.55 (1.790)	–0.5318 (1.588); 95% CI 0.87-0.20	–3.087 (81)	.003	
Control (n=29)		7.90 (1.944)	7.52 (1.755)	–0.386 (1.805); 95% CI 0.30-1.07	–0.149 (35)	.70	

^a^eHL: eHealth literacy.

^b^Some participant values are missing.

^c^HbA_1c_: glycated hemoglobin.

The mean baseline self-care behavior score (T0) was 79.83 (SD 27.17), and the mean increase after 3 months of the intervention (T2) was 81.09 (SD 26.70), showing a marginal and nonsignificant change (t_81_=511, *P*=.61). An unexpected result of this study was that the control group experienced an increase in self-care behavior scores (T0 mean 74.36, SD 26.69; T2 mean 84.06, SD 24.31; t_35_=4.503, *P*<.001). There were significant changes (*P*<.001) in the score of self-care behaviors between baseline and follow-up in the control group but not in the intervention group.

**Figure 1:**
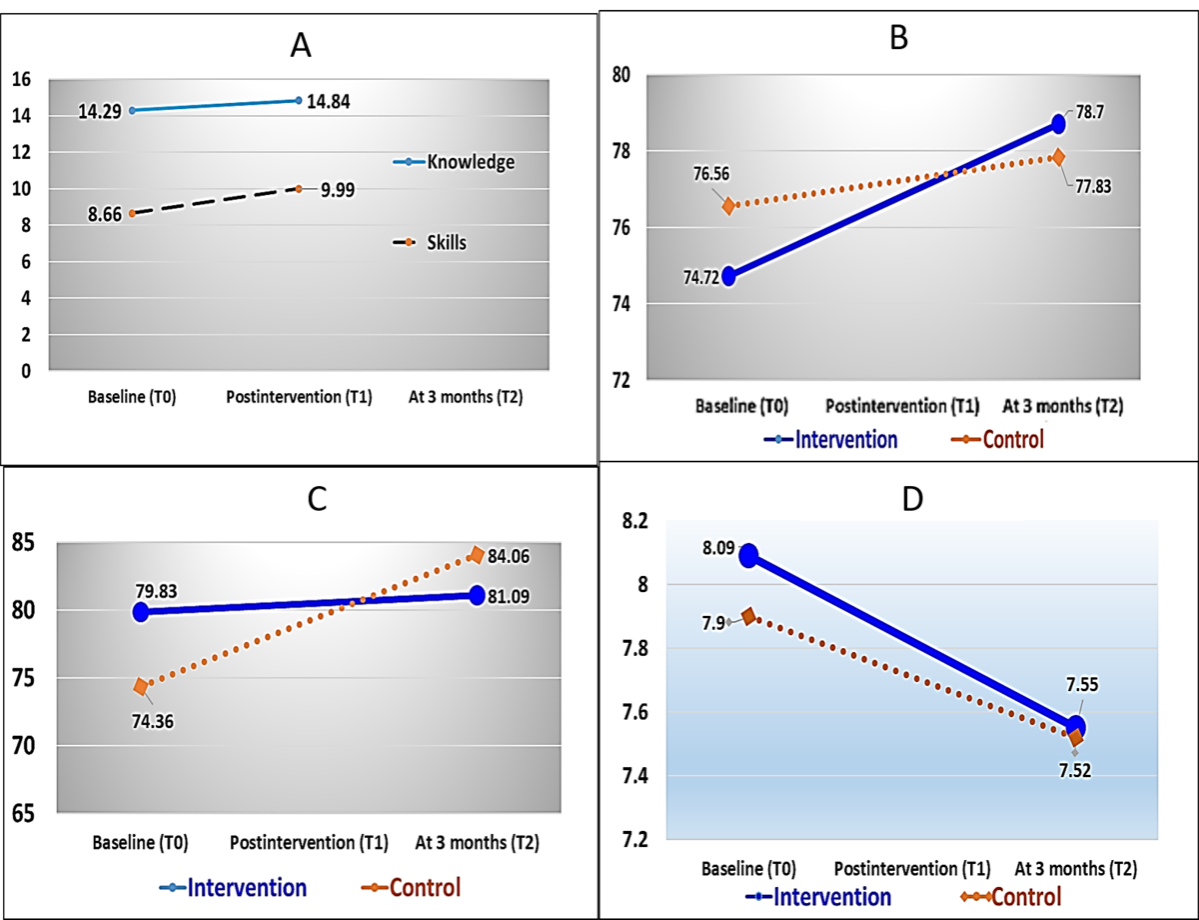
Line graphs (time × group) of mean differences at pretest (T0), posttest (T1), and 3-month follow-up (T2), using the metrics of (A) knowledge and skills, (B) mobile eHealth literacy (eHL), (C) self-care behavior, and (D) glycated hemoglobin (HBA_1c_).

The average HbA_1c_ levels at baseline in the intervention group and the control group were 8.09 (SD 2.11) and 7.90 (SD 1.94), respectively. Both groups exhibited high mean levels (normal <7.0 mg/dL) of HbA_1c_ at baseline. Three months later, the HbA_1c_ level in the intervention group and the control group was 7.55 (SD 1.79) and 7.52 (SD 1.76), respectively. However, the HbA_1c_ levels in the intervention group were slightly higher than those in the control group. The difference indicates a statistically significant reduction in HbA_1c_ levels in the intervention group (mean 0.53, SD 1.588, 95% CI 0.87-0.20, *P*=.003) compared with the reduction in the control group (mean 0.39, SD 1.805, 95% CI 0.30-1.07). Figure 1 displays a line graph illustrating the mean change at different time points, while Table 6 presents the chi-square values in the McNemar test. The test revealed a significant level in the experimental group (*P*=.004), indicating that the proportion of poor control and good control of HbA_1c_ in the interventional group before and after the intervention was significantly different (*P*=.004). The proportion of poor glycemic control was 61% (52/85) in the intervention group at the pretest, decreasing to 47% (40/85) in the posttest, which was significantly lower (*P*=.004). Furthermore, the proportion of HbA_1c_ changing from high to low (17%, 14/82) was greater than that of HbA_1c_ changing from low to high (2%, 2/85), indicating a positive intervention effect.

Table 7 indicates the regression coefficients of mHL and self-care behavior across 3 months. This suggests that the change of mHL in the intervention group (β=1.91, *P*=.047) and self-care behavior in the control group from T0 to T2 are significantly different (β=–8.21, *P*=.015).

**Table 6:** HbA_1c_^a^ change between pre- and posttest between the 2 groups.

Group/pre-HbA_1c_		Post-HbA_1c_^b^				McNemar test
		Low, n (%)	High, n (%)	Total, n (%)	*P* value	
**Intervention (n=85)**					.004	
	Low^c^	31 (36)	2 (2)	33 (39)		
	High^d^	14 (16)	38 (45)	52 (61)		
	Total	45 (53)	40 (47)	85 (100)		
**Control**					.38	
	Low	12 (41)	1 (3)	13 (45)		
	High	4 (14)	12 (41)	16 (55)		
	Total	16 (55)	13 (45)	29 (100.0)		

^a^HbA_1c_: glycated hemoglobin.

^b^Some participant values are missing.

^c^Low denotes HbA_1c_<7%.

^d^High denotes HbA_1c_≧7%.

**Table 7:** Between-group differences at pretest (T0) and at the 3-month follow-up (T2).^a^

Variables	Parameter estimates (β)					
	Preference health literacy	eHealth literacy	Mobile health literacy	Mobile eHealth literacy	Self-care behavior	HbA_1c_^b^
Intercept	15.19^c^	32.22^c^	30.42^c^	77.83^c^	84.06^c^	7.45^c^
Group	.12	–.43	1.29	.98	–3.53	.17
Time	.50	.42	.36	1.28	9.69^c^	–.35
Group×time	.09	.58	1.91^d^	2.61	–8.21^d^	–.19

^a^The control group is the reference group. The reference time is T0.

^b^HbA_1c_: glycated hemoglobin.

^c^*P*<.001.

^d^*P*<.05.

Among these coefficients, the mHL coefficient is positive, indicating that the increase in the intervention group is significantly higher than that in the control group (β=30.42, *P*<.001). By contrast, the self-care behavior coefficient is negative, signifying that the increase in the intervention group is significantly lower than that in the control group (β=–8.21, *P*<.05). Table 8 illustrates the effect sizes for changes in outcome measurements within the groups and between the 2 groups. Effect sizes fall within the range of small but noteworthy (*d*=0.2-0.5) and medium effect (*d*=0.5-0.8). Notably, the effect sizes for mobile eHL (*d*=0.49) and HbA_1c_ (*d*=0.33) in the intervention group were small but exceeded those in the control group (*d*=0.14 and *d*=0.16, respectively). Furthermore, the aggregated effect sizes between the 2 groups indicate that the average increase in mobile eHL and HbA_1c_ within the intervention group surpassed that of the control group (*d*=0.22 and *d*=0.14, respectively). Conversely, the collective effect size of self-care behavior within the intervention group was smaller than that of the control group (*d*=–0.28). The computed combined effect is negative because the augmented mean of the control group is greater than that of the intervention group.

**Table 8:** Effect sizes for change of outcome measurements within the groups and within-group change across the 2 groups.^a^

Variables		Intervention group (*d*)	Control group (*d*)	Intervention versus control (*d*)
**Mobile eHL^b^**		0.49	0.14	0.22
	Preference eHL	0.19	0.19	0.04
	eHL	0.18	0.10	0.01
	mHL^c^	0.38	0.07	0.33
Self-care behavior		0.09	0.66	0.28^d^
HbA_1c_^e^		0.33	0.16	0.14

^a^The effect size for mean differences of groups with unequal sample size within a pre- and posttest design.

^b^eHL: eHealth literacy.

^c^mHL: mobile health literacy.

^d^The order of the 2 groups was inverted to keep the Cohen *d* value positive.

^e^HbA_1c_: glycated hemoglobin.

## Discussion

### Principal Findings

The study had a dual focus: first, on the design and development of a mobile eHL program to facilitate health technology education, and second, on investigating its impact on patients’ outcomes. During the module design phase, the content was adapted from a valuable resource, specifically the NIA toolkit. These materials were then tailored to suit organizational websites and diabetes apps available in the Chinese language. The modules integrated interactive multimedia features and offered step-by-step guidance to facilitate health information education practice. Consequently, patients enhanced their literacy in utilizing health technology, enabling them to better manage their self-care.

The efficacy of a mobile eHL intervention for patients with diabetes was evaluated by analyzing both short-term outcomes (knowledge/skills) and long-term outcomes (mobile eHL, self-care behaviors, and HbA_1c_) at baseline, on the test day, and at the 3-month mark. The key finding of this study was that the intervention led to partial improvements in patient outcomes within the intervention group, encompassing knowledge, skills, mobile eHL, and HbA_1c_ levels.

### Comparison With Prior Work

Following the intervention, participants in the intervention group demonstrated a significant increase in knowledge and skills. This effect of the eHL intervention aligns with the findings of Xie [31], who observed a positive change among older adults when they were provided with eHL education, resulting in more positive attitudes toward the intervention. The enhancement of knowledge and skills in computer, web, and mobile technology observed after participants received the intervention aligns with findings from prior research [31,55].

While the results indicated that the change in the intervention group for the eHL subscale scores was nonsignificant, both the mHL subscale and the total mobile eHL scale scores demonstrated significant improvement. This discrepancy may be attributed to participants in the intervention group potentially possessing a higher level of comfort with using devices and internet browsing, which could explain the nonsignificant improvement in eHL. However, despite this, there was a noteworthy improvement in the mobile eHL and mHL scores.

These findings suggest that the mobile eHL acquired by participants through the intervention indeed improved their ability to utilize mobile and internet technology. Previous studies have pointed out that inadequate access to mHealth apps, designed to support patient self-management, often arises from a mismatch between the technology and the needs of patients or end users [13,53,57], particularly among individuals not well-versed in computer use and mobile skills [15,16,58]. Therefore, this study could emerge as an effective strategy to enhance patients’ comprehension of health technology.

Regarding the outcomes of HbA_1c_, the levels in the intervention group hovered around the borderline of the goal, slightly surpassing those of the control group. However, the difference in HbA_1c_ levels at the 3-month follow-up revealed a statistically significant reduction in the intervention group compared with the control group. According to the literature, determining the optimal glycemic target for patients with diabetes remains a subject of debate [59]. Typically, an HbA_1c_ level of 7% serves as a cutoff point, and a value below 7% is generally considered indicative of good control [50,60]. In our study, we used the 7% threshold, and according to our analytical results, some participants in the intervention group did not demonstrate lower levels of HbA_1c_ at follow-up. Other studies have made various attempts to enhance HbA_1c_. For instance, a team-based diabetes educational research study reported more significant improvement in HbA_1c_ levels after 12 months [61]. Therefore, a more extended intervention period may be worth considering.

However, in our study, an unexpected result emerged; specifically, the effect size result for self-care behaviors was only medium in the control group. The reason for this discordant result remains unclear and could be attributed to the absence of a randomized control trial design. Furthermore, the varying characteristics among participants, including their occupations, could contribute to the disparate findings. Another potential reason might be that a mobile eHL enhancement program could empower patients rather than correct their undesirable behaviors. Previous research has indicated that eHL does not have a direct effect on patient outcomes [42]. Hence, future studies may consider extending the duration of the study or intensifying the intervention further to explore potential impacts. Additional investigation should delve into how health information technology resources and diabetes apps are utilized following digital diabetes education.

Patient education stands as a crucial routine for health care providers. Technology plays a pivotal role as a self-care support and educational tool for both patients and health care providers, providing essential information in the pursuit of effective diabetes management. The mobile eHL intervention featured iPad-delivered literacy lessons, constituting an innovative and interactive multimedia module. This approach was developed to offer a structured method for involving patients in their care, ensuring universal access to health websites and diabetes apps. Our intervention signifies a transition in patient education from the conventional routine care model to a proactive digital approach. Here, mobile eHealth resources are customized to individual needs in diabetes self-care. While the intervention program may not routinely educate individuals about their illnesses and treatments, it has the potential to inspire health care providers to integrate mobile eHealth technology into patient education.

In summary, this study illustrated essential steps for instructing patients on accessing reliable health websites and diabetes apps. The mobile eHL education offered valuable insights into the development of a diabetes educational tool, providing a profound understanding of the necessity for digital diabetes education.

### Implications for Practice and Future Research

Technology, on its own, will not resolve enduring health disparities. Its effectiveness must be complemented by the active involvement of health care professionals, particularly in the context of patients with diabetes [11]. The utilization of mobile technology and eHealth in the field of chronic disease management is not a novel concept and has primarily been used for the collection and monitoring of physiological parameters in recent years. A plethora of health websites and mHealth apps continue to emerge, with varying degrees of involvement from health care experts [12,15,16]. Health care providers express concerns about potential changes in workload and the dynamics of consultations when integrating mobile eHealth technology into health care services [12]. The mobile eHL program encompasses a user-friendly educational toolkit created by our research team, aiming to mitigate workload concerns and facilitate the sustained use of advanced health technology. The outcomes of the mobile eHL intervention could potentially influence the attitudes of health care experts regarding their engagement with these advanced technologies. Educational modules can be perceived as a form of self-learning material for patients, requiring less assistance from health care professionals. Our findings imply that mobile mHL education has the potential to be integrated into chronic care delivery, allowing health care providers to play a facilitative role in the implementation of diabetes technology.

### Limitations

The real-world utilization of health technology among the intervention group following our intervention is a crucial aspect of the broader mobile eHealth research project. However, it is recommended that this be explored as a topic in future studies.

The study has limitations, including reliance on self-reports and a research design lacking random assignment to intervention or control groups. While a randomized controlled trial would be ideal, practical considerations, such as potential unacceptability to patients, render it infeasible. Our research team acknowledges some concerns in this regard. First, participants dedicated over 30 minutes to learning specific health-related websites and diabetes apps during the intervention. Additionally, they were tasked with demonstrating the steps of downloading apps onto their smartphones. Throughout enrollment, efforts were made to avoid jeopardizing the patient-health care provider relationship by ensuring that patients were not unduly pressured to learn and use technology. Second, some individuals exhibited technophobia, experiencing a noticeable fear of technology, particularly with computers or smartphones. This phenomenon seemed to be more prevalent among older adults. Further research could explore a trial randomly assigning participants to either a group education session or an individualized education session to delve deeper into these dynamics.

Finally, it is important to note that this study did not analyze information regarding other factors thought to elucidate the relationship between self-care behaviors and HbA_1c_ outcomes. Despite comprehensive data collection, the possibility remains that unmeasured confounders could influence the study’s results. While the effect size in our study is small, it remains noteworthy. It is crucial to recognize that the value of the effect size can be influenced by the sample size. Therefore, future studies are recommended to consider larger sample sizes. Despite the acknowledged limitations, this study has yielded meaningful results. Even though some variables did not exhibit significant improvement, they still exerted some impact on patient outcomes following the intervention. Furthermore, this study is the first to describe and compare the mobile eHL intervention among individuals with diabetes. This offers valuable insights into the eHL and mHealth app experiences of the diabetic population. The examination of various aspects of internet use has provided new information about the experiences, opinions, and attitudes of those with diabetes toward computers and the internet.

### Conclusions

This study revealed that the mobile eHL intervention positively influenced the familiarity of patients with T2D with health technology, consequently impacting their glycemic outcomes. The findings contribute to the existing body of knowledge concerning the design and adoption of digital patient education. A more precise understanding of the co-occurrence patterns of self-care behaviors would enhance the effectiveness of deploying technology resources to support chronic care. This study underscores the advantages of using well-structured modules and a multimodality approach for educating patients with T2D. This knowledge will be instrumental in shaping disease management, impacting not only clinical practice but also medical education.

## Appendix

Multimedia Appendix 1 The modules of mobile eHealth education.

Multimedia Appendix 2 Examples of screenshots of the first module of the mobile eHealth education.

Multimedia Appendix 3 Examples of screenshots of the second module of the mobile eHealth education.

Multimedia Appendix 4 The Questionnaire of Mobile eHealth Literacy.

Multimedia Appendix 5 The flowchart of the study.

## Abbreviations

eHEALS: eHealth Literacy Scale
eHL: eHealth literacy
HbA_1c_: glycated hemoglobin
KR-20: Kuder-Richardson Formula 20
mHL: mobile health literacy
NIA: National Institute on Aging
T2D: type 2 diabetes

## Data Availability

The data generated and analyzed during this study are available from the corresponding author upon reasonable request.
