# Supplementary material for: The Effect of Mobile eHealth Education to Improve Knowledge, Skills, Self-Care, and Mobile eHealth Literacies Among Patients With Diabetes: Development and Evaluation Study
Source: J Med Internet Res. 2023 Dec 6;25:e42497. doi: 10.2196/42497 (PMC10733817; doi:10.2196/42497)
Supplement: Multimedia Appendix 1 [file jmir_v25i1e42497_app1.docx]

|  | Session | Learning goals |
| --- | --- | --- |
| **First module: eHealth content** | | |
|  | Internet basics (NIA module #1)  Operation of health99 website (https://health99.hpa.gov.tw/) | 1. Learn basic computer terms and basic website terms. 2. Learn how to get to a website. 3. Learn how to explore a website. 4. Learn how to use a site map |
|  | Introduction to the Health99 website (NIA module #2)  (https://health99.hpa.gov.tw/) | 1. Use the Health Topics on the Health99 Home Page. 2. Find information in the Menu of the health topics. 3. Enlarge, view, close images, and view videos. 4. Search a health topic on Health99. 5. Navigate through several pages of a health topic. |
|  | Introduction to the Health and Exercise Association website (NIA module #2)  (https://www.exercise.org.tw) | 1. Use the Exercise Topics on the Exercise Website Home Page. 2. Find information in the Menu of the Exercise Website. 3. Navigate through several pages of a exercise topic, such as benefits and safety. 4. Find a right exercise video and images for yourself on Exercise Website. |
|  | Introduction to the Taiwan Food and Drug Administration (TFDA) website (NIA module #2)  (https://www.fda.gov.tw) | 1. Use the Drug Topics on the TFDA Home Page. 2. Find drug information in the Menu of the TFDA Website. 3. Navigate through several pages of a drug topic, such as drug effect. |
|  | Evaluating Health Websites (NIA module #9) | learn 9 indicators to recognize and locate:   1. Reliable health information websites such as web addresses end in “.gov or .org”. 2. The sponsor of a health website. 3. The purpose of a health website. 4. The authors of the health information. 5. The reviewers of the heath information. 6. The most recent update of the health information. 7. The privacy policy of a health website. 8. Clues about the accuracy of a website’s health information. 9. The contact information for a health website |
| **Second module: mHealth app content** | | |
|  | Mobile and app basics | 1. Learn basic mobile terms and basic app terms. 2. Learn how to goes online on mobile devices. 3. Learn how to download mobile apps from app stores. 4. Learn how to explore an app on websites. 5. Practice using screen touch. |
|  | Introduction to three Diabetes Apps developed by organizations /foundations/ commercial vendors. (in Chinese version)   1. Telecare app 2.0 2. TADE app 3. Health2Sync app | 1. Learn the searching terms related to diabetes. 2. Learn how to search and download diabetes apps by typing keywords. 3. Learn how to use a diabetes app, including register steps, type blood sugar, date, and time. 4. Find a right diabetes app you prefer to use. |
