# Supplementary figures and images for: The Effect of Mobile eHealth Education to Improve Knowledge, Skills, Self-Care, and Mobile eHealth Literacies Among Patients With Diabetes: Development and Evaluation Study
Source: J Med Internet Res. 2023 Dec 6;25:e42497. doi: 10.2196/42497 (PMC10733817; doi:10.2196/42497)

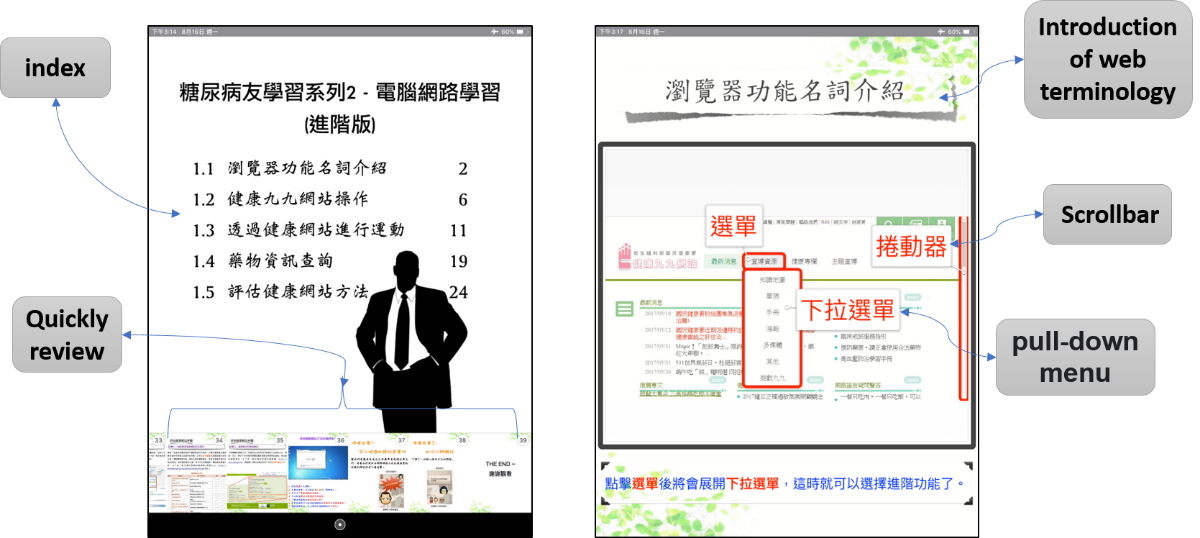

Supplement: Multimedia Appendix 2 [file jmir_v25i1e42497_app2.png]

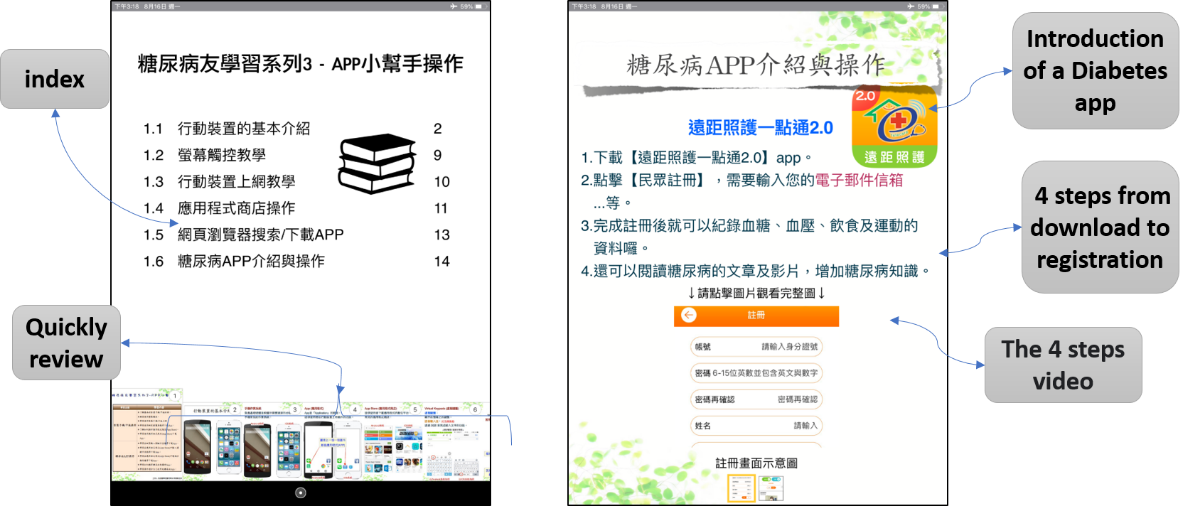

Supplement: Multimedia Appendix 3 [file jmir_v25i1e42497_app3.png]

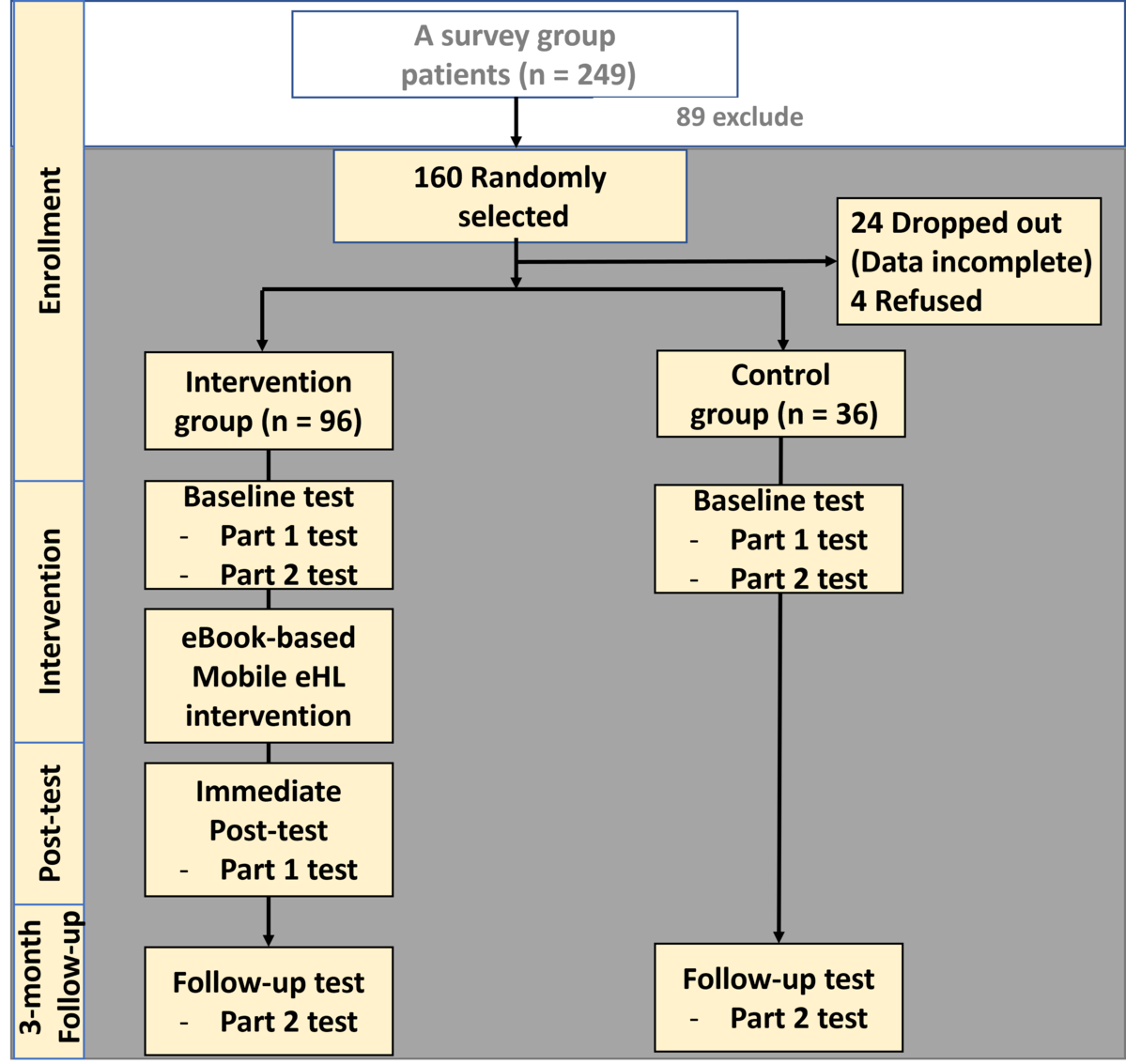

Supplement: Multimedia Appendix 5 [file jmir_v25i1e42497_app5.png]
